# Influence of Folate-Related Gene Polymorphisms on High-Dose Methotrexate-Related Toxicity and Prognosis in Turkish Children with Acute Lymphoblastic Leukemia

**DOI:** 10.4274/tjh.2016.0007

**Published:** 2017-06-01

**Authors:** Burcu Yazıcıoğlu, Zühre Kaya, Sezen Güntekin Ergun, Ferda Perçin, Ülker Koçak, İdil Yenicesu, Türkiz Gürsel

**Affiliations:** 1 Gazi University Faculty of Medicine, Department of Pediatric Hematology, Ankara, Turkey; 2 Gazi University Faculty of Medicine, Department of Genetics, Ankara, Turkey

**Keywords:** Methotrexate, Toxicity, prognosis, Folate-related gene polymorphisms

## Abstract

**Objective:**

High-dose methotrexate (HD-MTX) is widely used in the consolidation phase of childhood acute lymphoblastic leukemia (ALL), but the roles that polymorphisms in folate-related genes (FRGs) play in HD-MTX toxicity and prognosis in children with ALL are not understood. The aims of this study were to investigate the frequencies of polymorphisms in the genes for thymidylate synthase (TS), methionine synthase reductase (*MTRR*), and methylene tetrahydrofolate reductase (*MTHFR*) in Turkish children with ALL and to assess associations between these polymorphisms and HD-MTX-related toxicity and leukemia prognosis in this patient group.

**Materials and Methods:**

FRG polymorphisms were assessed by real-time polymerase chain reaction. Survival status, MTX levels, and toxicity data were retrieved from 106 patients’ charts.

**Results:**

The allele frequencies for the FRG polymorphisms were as follows: TS 2R 41.0%, 3R 57.0%, and 4R 2.0%; *MTRR* 66A 42.4% and 66G 57.6%; *MTHFR* 677C 59.3% and 677T 40.7%; and *MTHFR* 1298A 58.1% and 1298C 41.9%. At the 48th hour of HD-MTX infusion, serum MTX was significantly higher in patients who had TS 2R/3R/4R variants as compared to those with wild-type TS (p<0.05). No significant differences were detected with respect to event-free survival or toxicity between wild-type and other FRG variants.

**Conclusion:**

The frequencies of FRG polymorphisms in Turkish children with ALL are similar to those reported in other Caucasian populations. This is the first published finding of the TS 3R/4R variant in the Turkish population. The results indicate that HD-MTX can be tolerated by leukemic children with some polymorphic variants of FRG; thus, it may prevent future risk of leukemic relapse.

## Abstract (tr)

**Amaç:**

Yüksek doz metotreksat (YD-MTX) çocukluk çağı akut lenfoblastik lösemi (ALL) tedavisinin yoğunlaştırma fazında yaygın olarak kullanılmaktadır, ancak ALL’li çocuklarda YD-MTX’in toksisite ve prognozunda rol oynayan folat ilişkili genlerdeki (FİG) polimorfizmlerin rolü iyi bilinmemektedir. Bu çalışmanın amacı; ALL’li Türk çocuklarındaki timidilat sentaz (TS), metionin sentaz redüktaz (*MTRR*) ve metilen tetrahidrofolat redüktazı (MTHFR) kodlayan genlerdeki polimorfizm sıklığını araştırmak, bu hasta grubunda polimorfizmler ile YD-MTX’e bağlı toksisite ve lösemi prognozu arasındaki ilişkiyi değerlendirmektir.

**Gereç ve Yöntemler:**

FİG ilişkili polimorfizmler polimeraz zincir reaksiyonu ile incelenmiştir. Hastane dosyasından 106 hastanın yaşam durumu, MTX düzeyleri ve toksisite verileri kaydedilmiştir.

**Bulgular:**

FİG ilişkili polimorfizmlerin sıklığı sırasıyla TS 2R %41,0, 3R %57,0 ve 4R %2,0; *MTRR* 66A %42,4 ve 66G %57,6; *MTHFR* 677C %59,3 ve 677T %40,7; ve *MTHFR* 1298A %58,1 ve 1298C %41,9’dur. TS 2R/3R/4R varyant tipini taşıyan çocuklarda YD-MTX infüzyonunun 48. saatindeki serum MTX düzeyi yaban tipini taşıyanlardan daha yüksek oranda bulunmuştur (p<0,05). FİG ilişkili polimorfik varyantlar ile yabanıl tipleri arasında olaysız sağkalım ve toksisite yönünden anlamlı farklılık saptanmamıştır.

**Sonuç:**

ALL’li Türk çocuklarındaki FİG ilişkili polimorfizm sıklığı beyaz ırk popülasyonuna benzer oranda tespit edilmiştir. Türk popülasyonunda TS 3R/4R varyantı ilk kez bildirilmiştir. Sonuçlarımız FİG ilişkili bazı polimorfik varyantları taşıyan lösemili çocuklarda YD-MTX’in tolere edilebileceğine ve böylece gelecekteki relapsı önleyebileceğine işaret etmektedir.

## INTRODUCTION

Methotrexate (MTX) is a folate antagonist that impairs DNA synthesis and methylation reactions in cells. The metabolism of MTX is influenced by a number of polymorphisms in folate-related genes (FRGs) that encode the enzymes thymidylate synthase (TS), methionine synthase reductase (*MTRR*), and methylene tetrahydrofolate reductase (*MTHFR*) [1]. Currently, high-dose (HD) MTX is recommended for all patients with acute lymphoblastic leukemia (ALL) and for any patient with lymphoma or osteosarcoma [2,3,4]. However, there is no consensus on effective doses of HD-MTX in these patients. Recent research efforts have focused on determining the influence of different polymorphic enzyme variants on MTX toxicity and prognosis in children with ALL [5,6,7,8,9,10,11,12,13,14,15,16,17,18,19,20,21,22,23,24,25,26,27,28,29,30,31,32,33]. Some have reported that several polymorphic variants of FRGs may be linked to relapse and HD-MTX-related toxicity in children with leukemia [5,6,7,8,9,10,11,12,13,14,15,16,17,21,22,23,24,25,26,27,28,29], whereas others have found that this treatment is protective against leukemia and there is no association between FRGs and toxicity [18,19,20,30,31,32,33].

The frequencies of these gene polymorphisms vary widely among different races, ranging from 5% to 50% depending on the enzyme type [21,24,25,26,34,35]. To date, only two studies have examined the link between polymorphisms in *MTHFR* genes and leukemia in Turkish children [3,36]. The aims of this study were to determine the frequencies of TS, *MTRR*, and *MTHFR* polymorphisms in Turkish children with ALL and to evaluate possible associations with HD-MTX toxicity and survival in this patient group.

## MATERIALS AND METHODS

The study involved 106 children with ALL who were treated with the ALL-Berlin-Frankfurt-Munster (BFM) 95 protocol at our clinic between 1998 and 2014. The study protocol was approved by Gazi University Faculty of Medicine review board. Patients were assigned to risk groups and treated according to the ALL-BFM 95 protocol, as described previously [37].

All patients received four courses of 5 g/m^2^ MTX together with 25 mg/m^2^ mercaptopurine during consolidation phases. Fifteen patients treated prior to 2003 had received intermediate doses of ARA-C (ID-ARA-C) at 200 mg/m^2^ with HD-MTX+MP in the consolidation phase. Serum MTX levels were measured at the 24^th^, 36^th^, 42^nd^, and 48^th^ hours of infusion. Effective serum MTX levels according to the BFM 95 protocol were defined as ≤150 µmol/L, ≤3 µmol/L, ≤1 µmol/L, and ≤0.4 µmol/L for the 24^th^, 36^th^, 42^nd^, and 48^th^ hours, respectively. Serum MTX levels and treatment-related toxicity data were retrieved from the patients’ charts retrospectively. National Cancer Institute criteria were used to evaluate toxicity.

### Analysis of Folate-Related Genes

While TS and *MTRR* gene polymorphisms were analyzed in all 106 patients, findings for *MTHFR* polymorphisms were only recorded for the 43 patients with complete clinical data (i.e. significant data were missing for the remaining patients).

### DNA Extraction

DNA was isolated from a blood sample from each patient according to the NucleoSpin^®^ blood kit protocol (Macherey-Nagel, Düren, Germany). The concentration and quality of DNA were analyzed by spectrophotometer (NanoDrop ND 1000, Thermo Fisher Scientific, Waltham, MA, USA).

The primers used to detect the *MTRR* gene 66 A>G polymorphism were 5’-AAGGCCATCGCAGAAGACAT-3’ and 5’-CCATTGAACAAACACATTTCTG-3. The primers used to detect the tandem repeat sequence in the enhancer region (TSER) of the TS gene were 5’-AACTGTGCTGCTGGCTTAGAGAA-3’ and 5’-ATGTCGGACTCTCCACTGCG-3’.

To identify the *MTRR* gene 66 A>G polymorphism, a 220-bp target gene region amplified using specific primers was incubated with AflIII restriction enzyme overnight at 37 °C. The resulting product was loaded on 3% agarose gel and subjected to electrophoresis. Examination of the bands on the gel revealed a 220-bp band for the AA genotype and 203- and 17-bp bands for the GG genotype.

To identify TSER polymorphism of the TS gene, the amplified products of the primers above were loaded on 3% agarose gel and subjected to electrophoresis. A 578-bp band indicated the 2R/2R genotype, a 606-bp band and a 578-bp band indicated 2R/3R, a 606-bp band indicated 3R/3R, and a 634-bp band and a 606-bp band indicated 3R/4R.

### Real-Time Polymerase Chain Reaction

The *MTHFR* mutations of C677T and A1298C were amplified and detected by TaqMan probes using a real-time polymerase chain reaction (PCR) kit (SNP, Biotech, Ankara, Turkey). For the procedure, 20.5 µL of master mix and 0.3 µL of hot-start Taq DNA polymerase were added to a PCR tube, and 4.5 µL of the patient’s DNA suspension (100 µL) was then added. The following PCR program was performed: an initial denaturation step at 95 °C for 10 min, followed by 32 cycles of denaturation at 95 °C for 15 s, and annealing at 60 °C for 1 min. Allelic discrimination was facilitated by software analysis of the fluorescence data.

### Statistical Analysis

Data were statistically analyzed using SPSS 15.0. Genotype frequencies of the *TS, MTRR*, and *MTHFR* polymorphisms in FRGs were compared with previously reported findings for these enzymes in Turkish populations [3,34,35,36]. Differences between groups were analyzed using the Mann-Whitney U test. The chi-square test was used to analyze categorical data. Survival rates, including event-free survival (EFS) and overall survival (OS), were investigated using Kaplan-Meier analysis. Events were defined as relapse or death from any cause. OS was defined as time from initiation of treatment to death or the date of the last follow-up. Values of p<0.05 were considered statistically significant.

## RESULTS

The demographic features of 106 children with ALL are shown in Table 1.

### Genotype and Allele Frequencies of Folate-Related Genes

The results for allele frequencies of polymorphisms in FRGs were TS 2R 41.0%, 3R 57.0%, and 4R 2.0%; *MTRR* 66A 42.4% and 66G 57.6%; *MTHFR* 677C 59.3% and 677T 40.7%; and *MTHFR* 1298A 58.1% and 1298C 41.9% (Table 2).

### Serum Methotrexate Levels

Table 3 shows the serum MTX levels at different time points for the groups of patients with *TS, MTRR*, and *MTHFR* polymorphic variants. At the 48^th^ hour of HD-MTX infusion, serum MTX was significantly higher in patients who had TS 2R/3R/4R variants as compared to those with wild-type TS (p<0.05). There were no such statistical differences at the other time points. There were also no statistically significant differences between serum MTX levels at other time points for each of the polymorphic variants of the FRGs assessed.

### Toxicity Evaluation

After a total of 424 HD-MTX treatment courses in the 106 cases, grade III/IV severe anemia developed in 8.7% of the patients, leukopenia in 23.9%, neutropenia in 34.8%, and thrombocytopenia in 2.2%. Grade III/IV severe hepatic toxicity was recorded in 4.3% of the patients following HD-MTX, and renal toxicity in 11.3%. There were no significant differences between the respective wild-type groups and other FRG variants regarding hematologic and nonhematological toxicities (p>0.05). The toxicity findings for the 106 patients are shown in Table 4. Only one child with MTX encephalopathy carried the GG variant for *MTRR*, TT for *MTHFR* 677, and 2R/3R for TS. This patient rapidly recovered with aminophylline administration after 72 h of HD-MTX treatment. No severe mucositis was observed in patients who received only HD-MTX in the ALL-BFM 95 protocol, whereas grade III-IV mucositis had been observed previously in 15 (14.1%) of 106 patients who had received HD-MTX together with intermediate-dose ARA-C in the ALL-BFM 95 protocol in our clinic.

### Survival Status

**Figure 1:**
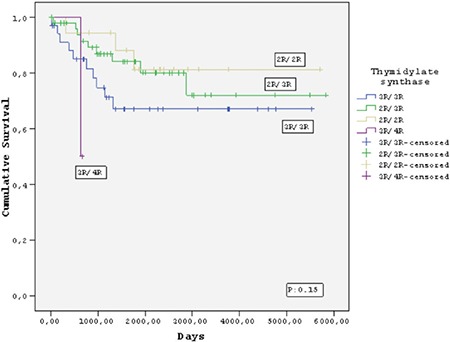
Kaplan-Meier estimate of event-free survival of the patients who carried thymidylate synthase polymorphisms.

**Figure 2:**
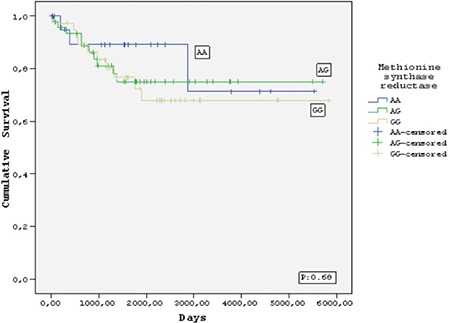
Kaplan-Meier estimate of event-free survival of the patients who carried methionine synthase reductase polymorphisms.

**Figure 3:**
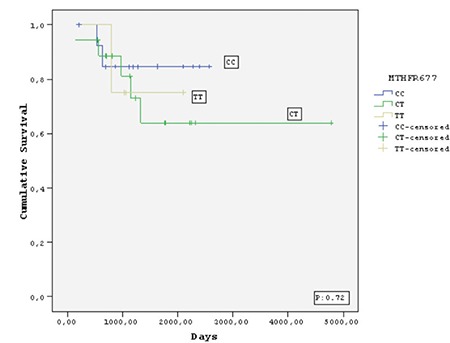
Kaplan-Meier estimate of event-free survival of the patients who carried methylene tetrahydrofolate reductase 677 polymorphisms. MTHFR: Methylene tetrahydrofolate reductase.

**Figure 4:**
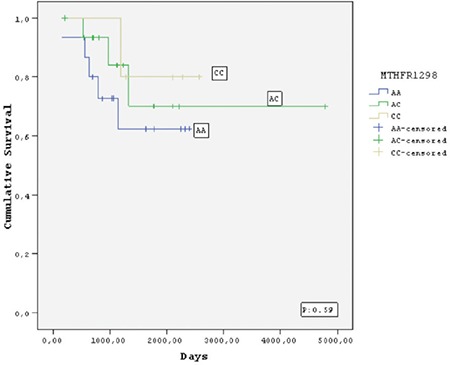
Kaplan-Meier estimate of event-free survival of the patients who carried methylene tetrahydrofolate reductase 1298 polymorphisms. MTHFR: Methylene tetrahydrofolate reductase.

Twelve (11.3%) of the 106 children died, 8 (7.6%) due to relapse or refractory disease and 4 (3.7%) due to infections during follow-up (median 58 months). No deaths occurred in patients who received HD-MTX during consolidation therapy. Of the 106 patients who achieved complete remission, 19 (17.9%) relapsed after a median of 26.2 months. The relapse rates were 16/19 (84.2%) for TS genotypes (2R/3R, 3R/3R, and 3R/4R), 17/19 (89.4%) for *MTRR* genotypes (AG and GG), 6/19 (31.5%) for *MTHFR* 677 genotypes (CT and TT), and 2/19 (10.5%) for *MTHFR* 1298 genotypes (AC and CC). However, there were no significant differences with respect to relapse rates, EFS, or OS between the groups with and without polymorphic variants of FRG (Figures 1,2,3,4).

## DISCUSSION

MTX is a key component of consolidation and maintenance treatment for childhood ALL [2]. However, some patients cannot tolerate HD-MTX, and in these cases the treatment can cause toxicity and discontinuation of chemotherapy, which may increase relapse risk in a small number of patients [5,8,23,24,25,29]. Our study is the first to have investigated the frequencies of FRG polymorphisms and to have assessed associations between these polymorphisms and HD-MTX-related toxicity and outcomes in Turkish children with ALL. The respective frequencies of the TS 2R/2R, 2R/3R, and 3R/3R variants in our patients were 17.0%, 48.1%, and 33.0%. While these are not different from findings in the healthy Turkish population and other Caucasian populations [34,38], higher frequencies for the TS 3R/3R variant (66% to 76%) and lower rates for other variants (2R/2R 1% to 3%; 2R/3R 22% to 29%) compared to the Turkish population were reported in a study of Indonesian children and in other reports from Japanese and Chinese populations [21,34]. We detected the TS 3R/4R variant in only two patients, and ours is the first published finding of this variant in the Turkish population. One of these patients died due to cardiac and hepatic toxicity after salvage chemotherapy for relapse. The other child was diagnosed with high-risk leukemia, was treated with allogeneic stem cell transplantation, and is currently in remission. The same genetic pattern was previously described in 20 leukemic children who were receiving the Children’s Cancer Group-1891 protocol; 7 (35%) of these patients relapsed and the TS 3R/4R genotype was associated with significantly greater relapse risk in that study [23]. Our limited data confirm the previous observation that some patients with the TS 3R/4R genotype who achieve limited benefit from chemotherapy alone should ultimately undergo transplantation.

The frequencies of the *MTRR* AA, AG, and GG variants in our sample were 20.8%, 43.4%, and 35.8%, respectively. Similarly, the corresponding rates reported for healthy vs. leukemic children from Slovenia were 18.2% vs. 22.1%, 52.7% vs. 52.9%, and 29.1% vs. 25.0% [38]. The only previous investigation of *MTRR* polymorphism frequency in the Turkish population was conducted in children with stroke [35]; however, the frequency of homozygous GG variant (4%) for this enzyme in that study was lower than we observed in ours. Interestingly, one of our patients with the *MTRR* GG variant developed MTX encephalopathy. In our pediatric ALL patients, the frequencies of the *MTHFR* 677 CC, CT, and TT variants were 48.8%, 41.8%, and 9.3%, respectively, and the frequencies of the *MTHFR* 1298 AA, AC, and CC variants were 51.2%, 37.2%, and 11.6%, respectively. These results are comparable to those previously reported for children with ALL in the Turkish population [3] and other Caucasian populations [39].

There are conflicting results regarding the roles of the *TS, MTRR*, and *MTHFR* gene polymorphisms in leukemia prognosis [5,8,9,15,18,23,24,25,26,27,28,32,33,38]. Some studies have indicated that these variants play protective roles [32,33,38], whereas others have shown that they are linked to increased rates of relapse and drug resistance [5,6,7,8,9,15,18,23,24,25,26,27,28]. Among these gene polymorphisms, the higher enzyme activity of the TS 3R/3R variant led to the diminished MTX effect and enhanced drug resistance. A study performed at the Dana Farber Institute revealed that 32 of 205 children with ALL who were followed for 12 years developed relapse and/or died, and most of these children had the TS 3R/3R variant [24]. Similarly, an investigation of 246 children with ALL at the St. Jude Medical Center demonstrated that in high-risk cases, the TS 3R/3R polymorphism was significantly associated with development of relapse [25]. Another study performed by the BFM group in Germany investigated 40 ALL patients with relapse who received BFM 86-90 chemotherapy and found no significant difference in EFS between the TS 3R/3R and TS 2R/3R variants [26]. The Dana Farber and St. Jude centers applied MTX at single dosages of 4 g/m^2^ and 2 g/m^2^, respectively, whereas the German BFM group applied a cumulative dose of MTX with 5 g/m^2^ administered per cycle and repeated four times with 2-week intervals [24,25,26]. Thus, it has been suggested that the higher MTX dosage applied by the German group overcame the enhanced enzymatic activity of the TS 3R/3R genotype and reached sufficient therapeutic concentrations [26]. In accord with these data, we observed no significant differences in EFS between patients with or without polymorphic variants of TS and other enzymes, and this finding supports the effect of HD-MTX in our study.

Studies have yielded contradictory results regarding associations between HD-MTX-related toxicity and FRG polymorphisms in children with ALL [3,9,10,11,12,13,14,15,16,17,18,19,20,27,28,29,30,31]. Kantar et al. [3] reported that the *MTHFR* A1298C polymorphism caused severe hematological toxicity in patients with higher serum MTX levels, specifically anemia (62.5% of 37 cases), thrombocytopenia (51.5%), and aspartate aminotransferase elevation (11.8%). In contrast, a metaanalysis of all 14 studies on MTX suggested that less toxic effects were seen in cases with the *MTHFR* A1298 C polymorphism [14]. Most of these studies indicated that the *MTHFR* C677T gene polymorphism causes oral mucositis, myelosuppressive effects, and liver, intestinal, and skin toxicities, and also leads to an increased relapse rate in children with ALL [6,7,8,9,10,11,12,13,14,15,16,17,18,19,22]. Only one report from Turkey has not confirmed this association [3]. In contrast to other research, we observed a somewhat higher rate of renal toxicity in patients who carried the *MTHFR* 677CT/TT genotypes compared to wild-type *MTHFR* enzymes [3,11,12,13,14,22,23]. Nevertheless, our previous report indicated that when severe renal toxicity occurs shortly after HD-MTX administration in children with leukemia, it resolves almost completely with time [37]. In addition to these findings, severe mucositis was also reported in other studies of children with ALL who carried *MTHFR* 677TT and *MTRR* 66GG [22,30]; however, neither of these publications specified whether HD-MTX was used alone or in combination with ARA-C. We observed lower frequencies of severe mucositis only in patients who carried TS 2R/2R, *MTHFR* CT/TT, and *MTRR* AA genotypes and received HD-MTX together with ID-ARA-C; however, in our clinic we have not used ARA-C since 2003 because research has indicated that this treatment has no effect on relapse rate [40]. Of three recent studies that examined the influence of TS polymorphism on chemotherapy toxicity, one revealed no significant abnormality and the others indicated that TS 2R and 3R allele polymorphisms were significantly associated with lower frequencies of leukocytopenia, thrombocytopenia, and peripheral neuropathy [6,23,29]. In accord with these findings, none of our patients with polymorphic variants of FRGs developed significant toxicities despite the fact that those with TS 2R/3R/4R variants had higher MTX levels at the 48-h time point. The conflicting results among these studies may be related to differences in MTX dosages, toxicity grades, or drug combinations. The small sample size of our study population is the main limitation of the present findings. We observed no significant differences between wild-type and other FRG variants with respect to toxicity or relapse rate, but further investigations with larger patient numbers are needed.

## CONCLUSION

In conclusion, the frequencies of the *TS, MTRR*, and *MTHFR* polymorphisms in Turkish children with ALL are similar to those reported for other Caucasian populations. Our study is the first published finding of the TS 3R/4R variant in the Turkish population. Our results indicate that HD-MTX can be tolerated by leukemic children with some polymorphic variants of FRGs and thus it may prevent future risk of leukemic relapse.

## Figures and Tables

**Table 1:**
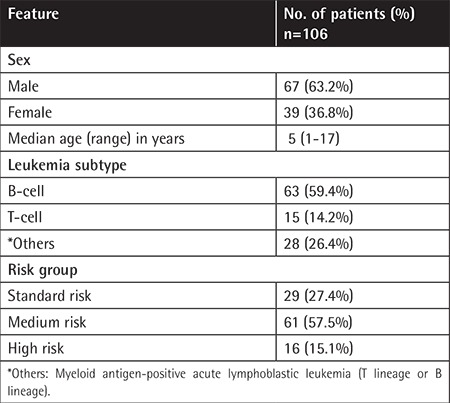
Demographic features of the pediatric patients with acute lymphoblastic leukemia.

**Table 2:**
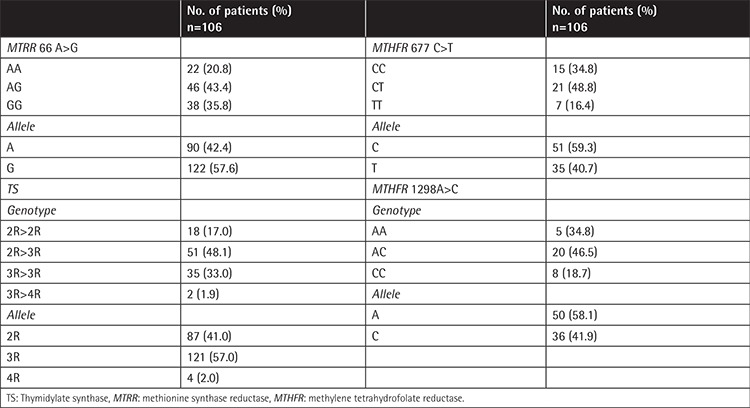
Genotype and allele frequencies for folate-related gene polymorphisms in the 106 children with acute lymphoblastic leukemia.

**Table 3:**
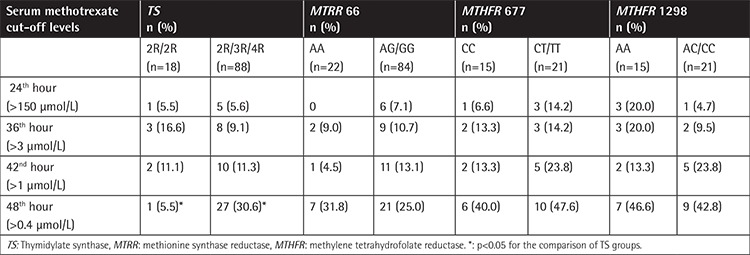
Serum levels of methotrexate at different infusion times with the patients grouped by folate-related gene polymorphisms.

**Table 4:**
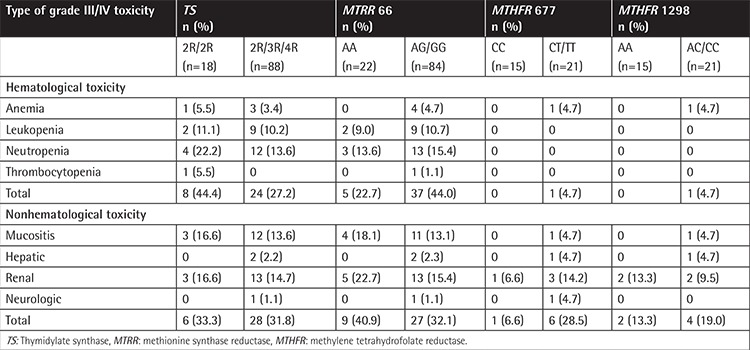
Comparison of grade III/IV toxicity findings with the patients grouped by folate-related gene polymorphisms.
